# Effects of Calorie Restriction With and Without Strength, Endurance or Mixed Training on Fat‐Free and Skeletal Muscle Mass in Overweight or Obese Individuals—A Systematic Review With Pairwise Meta‐Analysis and Network Meta‐Analysis of Randomized Controlled Studies

**DOI:** 10.1111/dom.70873

**Published:** 2026-05-17

**Authors:** Magdalena Deller, Jan Weiershaus, Steffen Held, Christian Brinkmann

**Affiliations:** ^1^ Department of Preventive and Rehabilitative Sport Medicine Institute of Cardiovascular Research and Sport Medicine, German Sport University Cologne Cologne Germany; ^2^ IST University of Applied Sciences Düsseldorf Germany

**Keywords:** caloric restriction, calorie restriction, diet, exercise, fat‐free mass, lean body mass, skeletal muscle, training

## Abstract

**Aims:**

Calorie restriction (CR) reduces both fat mass and fat‐free mass (FFM), particularly skeletal muscle mass (SMM), which is essential for cardiometabolic health and preventing frailty. This study compares CR alone versus CR plus exercise (EX) on FFM and SMM in overweight/obese patients and examines the effects of different training modes.

**Methods:**

A systematic literature search was conducted in the PubMed and Web of Science databases. After screening, 34 randomized controlled trials were included in the final analyses, involving a total of 1455 participants.

**Results:**

A random‐effects model showed a significant effect of EX on FFM, with a pooled mean difference favouring CR combined with EX over CR alone (mean difference (MD) = +0.87 kg; 95% confidence interval (CI): +0.59, +1.16 kg; *p* ≤ 0.001). On average, EX prevented nearly half of FFM loss (45.7%). While subgroup testing found no significant differences between training modes, point estimates suggested greater benefits when the intervention included strength training. An additional network meta‐analysis, conducted to account for the repeated use of the same control group in multi‐arm studies, revealed that mixed training yielded the largest effect (MD = +1.20 kg, 95% CI: +0.67, +1.73 kg; *p* < 0.001, P‐score = 0.93), followed by strength training (MD = +0.83 kg, 95% CI: +0.17, +1.49; *p* = 0.013; P‐score = 0.66), while endurance training fell just short of statistical significance (MD = +0.51 kg, 95% CI: −0.04, +1.05; *p* = 0.067; P‐score = 0.41). Only two studies reported SMM, both indicating muscle‐preserving effects of EX.

**Conclusions:**

Exercise training is very effective in reducing FFM loss during CR. The present study provides valuable new insights for optimizing weight loss strategies.

**Trail Registration:**

CRD420251058428

**Abbreviations**
BIA: bioelectrical impedance analysis
CR: calorie restriction
DXA: dual‐energy x‐ray absorptiometry
FFM: fat‐free mass
GIP: glucose‐dependent insulinotropic polypeptide
GLP‐1: glucagon‐like peptide 1
HIIT: high‐intensity interval training
LBM: lean body mass
MD: mean difference
MRI: magnetic resonance imaging
PICO: patient‐intervention‐comparison‐outcome
RCT: randomized controlled trial
RM: repetition maximum
SD: standard deviation
SE: standard error of the mean
SMD: standardized mean difference
SMM: skeletal muscle mass

## 1 Introduction

Calorie restriction (CR)‐induced weight loss is a well‐established strategy for improving cardiometabolic health in individuals who are overweight or obese [1, 2]. This effect is primarily driven by the reduction of dysfunctional fat mass (particularly visceral adipose tissue and ectopic fat in the liver, heart, skeletal muscle or pancreas) which releases pro‐inflammatory cytokines and fatty acids that contribute to cardiovascular complications and insulin resistance [3].

CR consistently also leads to losses in fat‐free mass (FFM), particularly skeletal muscle mass (SMM) [4]. Although the ratio of fat mass to skeletal muscle mass usually improves during CR [5], the loss of SMM remains critical for several reasons. Skeletal muscle is an important regulator of glucose homeostasis, accounting for 80% of postprandial glucose uptake [6]. Furthermore, muscle‐derived secretory proteins, known as myokines, mediate interactions between skeletal muscle and other organs (muscle‐organ cross‐talk) with numerous health‐promoting effects [7]. Loss of SMM may also increase the risk of frailty and impair physical function [8]. Therefore, minimizing muscle mass loss during CR is advisable.

In recent years, incretin‐mimetic therapies such as glucagon‐like peptide 1 (GLP‐1) and dual GLP‐1/glucose‐dependent insulinotropic polypeptide (GIP) receptor agonists have transformed the management of obesity and diabetes [9]. These agents induce substantial weight loss, among others, through appetite suppression and the resulting CR [10]. However, similar to non‐pharmacological CR, weight loss induced by incretin mimetic therapy is also accompanied by varying degrees of SMM loss [11].

Structured exercise is widely recommended to counteract declines in FFM and SMM during CR [12]. A previous meta‐analysis by Sardeli et al. [13] demonstrated that resistance training can preserve most of the FFM otherwise lost during CR in older adults, highlighting the potential of targeted training interventions to maintain musculoskeletal health. No recent meta‐analysis has focused exclusively on studies with pairwise (head‐to‐head) comparisons between CR and CR + exercise training in overweight/obese individuals. The primary aim of the present systematic review and meta‐analysis is to evaluate the effectiveness of different exercise modalities in preserving FFM and SMM during CR in this particular patient group. These results are particularly relevant, as the choice of training modality may substantially influence muscle preservation during both non‐pharmacological and pharmacological weight loss interventions.

## 2 Methods

### 2.1 Literature Search Strategy

The review was registered in PROSPERO (CRD420251058428). A literature search was conducted in accordance with PRISMA (Preferred Reporting Items for Systematic Reviews and Meta‐Analysis) guidelines [14] using the PubMed and Web of Science databases (original search: 08.06.2025). The full search strings are available in the appendix (File S1). As FFM and lean body mass (LBM) are often used interchangeably and, according to recent studies, represent the same chemical body components, only the term FFM is used throughout this review [15].

### 2.2 Eligibility Criteria

Eligibility was defined using a pre‐specified Patient‐Intervention‐Comparison‐Outcome (PICO) framework. Studies were included if they were randomized controlled trials (RCTs) with a clearly identifiable randomization method, enrolling adults aged ≥ 18 years with a mean body mass index (BMI) ≥ 30 kg/m^**2**^ (either within each intervention group or overall if group‐specific values were not available). Participants were excluded if they had internal medical conditions other than those associated with metabolic syndrome. Eligible trials had to compare a CR intervention (CR alone) with the same intervention combined with a structured exercise programme (CR + exercise), over ≥ 4 weeks. Interventions were required to target CR (with or without pharmacotherapy) and include clear instructions for reduced food/calorie intake. The CR‐only group was not permitted to receive additional physical activity recommendations. Exercise interventions were required to consist of structured strength, endurance or mixed training (combined strength and endurance training or functional training aimed at improving strength and cardiorespiratory fitness), with sufficient detail provided on the training programme. Studies had to report FFM, LBM or SMM both pre‐ and post‐intervention. Pre‐ and post‐values, along with the number of participants in each group pre‐ and post‐intervention, had to be reported, regardless of whether analyses were conducted on an intention‐to‐treat or per‐protocol basis. Only full‐text articles published in English were eligible. Studies were included only if final measurements were completed within 1 week post‐intervention.

### 2.3 Study Selection and Data Collection

The retrieved records were imported into *Rayyan* (https://www.rayyan.ai) [16]. Two authors (MD and JW) independently screened all records by title and abstract after removing duplicates, followed by full‐text screening. Any disagreements were resolved through discussion with a third reviewer (CB) until consensus was reached.

The extracted data included first author, year of publication, country, total sample size, number of groups, group‐specific sample sizes, sex and age of participants, details of the exercise interventions, details of the dietary interventions, methods used to assess FFM or SMM, reported comorbidities, and whether statistical analyses were conducted per protocol or intention‐to‐treat. Pre‐ and post‐intervention data for FFM and total body mass were also extracted.

If not reported, change‐from‐baseline values were calculated by subtracting the mean_pre_ value from the mean_post_ value (Δ = mean_post_ − mean_pre_) for each group (CR and CR + exercise). If not reported, standard deviations (SDs) for change scores were calculated from the reported standard errors (SEs) of change scores, if provided and greater than zero, using the following formula: SD = SE × √n. If this was not possible, they were imputed using SDs from studies with similar characteristics included in the analysis (suitable studies were selected based on the following criteria and order: same control conditions/training mode, same body composition measurement method, similar number of participants, and similar number of total training sessions), in accordance with the Cochrane Handbook 5.1. (https://handbook‐5‐1.cochrane.org, chapter 16.1.3.2 “Imputing standard deviations for changes from baseline”) [17]. When means and SDs or SEs were presented only graphically, WebPlotDigitizer (version 4, Free Software Foundation, Boston, MA, USA) was used to extract the data (in one study). Where available, data were extracted from per‐protocol analyses (*n* = 27 studies) (they were otherwise extracted from intention‐to‐treat analyses [*n* = 7 studies]).

If a study included multiple study arms in addition to a control group (e.g., different exercise training interventions), all relevant study arms were included and compared separately to the control group in the pairwise meta‐analysis.

### 2.4 Quality Assessment of Randomized Controlled Trials

The methodological quality of the included studies was assessed using the PEDro (Physiotherapy Evidence Database) scale [18]. Two authors (MD and CB) independently rated the studies and any disagreements on individual items were resolved by a third reviewer (SH) who made the final decision. The PEDro scale consists of 11 items, in which Criteria 2–9 evaluate randomization and internal validity, while Criteria 10–11 assess whether results are statistically replicable. Criterion 1 addresses external validity and is not included in the total score. Higher PEDro scores indicate better quality. Since blinding of instructors and participants in exercise intervention studies is challenging, though sometimes feasible using sham interventions, items 5–6 were rated accordingly. Total scores of 3 or less indicated “poor” quality, scores of 4–5, “fair”, 6–8, “good”, and scores of 9–10 “excellent” quality.

In addition, study quality was assessed by the same researchers using the domain‐based “Risk of Bias 2” (RoB2) evaluation tool [19]. The five RoB2 domains included ‘bias arising from randomization process’, ‘bias due to deviations from intended interventions’, ‘bias due to missing outcome data’, ‘bias in measurement of the outcome’ and ‘bias in selection of the reported results’, assessed using signalling questions. The overall risk of bias was categorized as “low risk”, “some concerns” or “high risk”.

### 2.5 Statistical Analyses

Mean differences (MDs) and corresponding 95% confidence intervals (CIs) were calculated as measures of treatment effectiveness. MDs were coded, with positive values indicating higher outcomes for CR + exercise training compared with CR alone. Separate meta‐analyses were conducted for FFM and body mass, with additional subgroup analyses to explore key moderators. These subgroups included mode of training (strength, endurance or mixed) and sex (females, males or mixed). For each meta‐analysis, a random‐effects model was used as the primary analysis to account for variability both within and between studies [20]. Heterogeneity was evaluated using the I^2^ statistic and interpreted as low (≤ 50%), moderate (50%–75%), or high (> 75%) [21]. To assess the influence of individual studies, a leave‐one‐out analysis was performed, in which each study was sequentially removed, and overall intervention effects and CIs were re‐estimated.

Furthermore, meta‐regression analyses were conducted to examine whether the total number of training sessions moderated the intervention effect on FFM or body mass.

To examine whether the magnitude of weight loss explained heterogeneity in FFM outcomes, additional meta‐regression analyses with restricted maximum likelihood estimation were performed. The MD in FFM served as the dependent variable, while body mass change in the control arm, body mass change in the intervention arm, and the between‐group MD in body mass were tested as separate moderators.

Publication bias was evaluated through visual inspection of funnel plots. Additionally, Egger's regression test was performed for each meta‐analysis to statistically assess funnel plot asymmetry and potential small‐study effects [22]. The primary analytical approach was a pairwise meta‐analysis, designed to evaluate direct comparisons between intervention conditions (CR + exercise training) and CR alone (control). This approach also aligned with the prespecified subgroup analyses and meta‐regressions focusing on these direct contrasts. However, several included studies used multi‐arm designs with shared control groups. In an exclusively pairwise framework, such studies require additional handling decisions, such as using control groups multiple times or combining intervention arms. Therefore, while pairwise meta‐analysis was retained as the primary approach, a network meta‐analysis was additionally conducted on the same dataset to preserve the full study structure and integrate both direct and indirect evidence across interventions.

The network meta‐analysis was implemented as a frequentist random‐effects model using the netmeta package in R [23]. Separate networks were fitted for FFM and body mass, with CR alone defined as the reference (control) node, and MDs (in kg) used as the common outcome metric. SEs from multi‐arm trials were adjusted using the established correction for within‐study correlation. The between‐study variance (τ^2^) was estimated with the DerSimonian and Laird estimator, and I^2^ was used to quantify heterogeneity [24]. Inconsistency between direct and indirect evidence was assessed by comparing heterogeneity within designs to heterogeneity between designs. Relative treatment effects are reported as mean differences with 95% CIs. In addition, P‐scores were calculated for each intervention under the random‐effects model to rank treatments, with higher values indicating more favourable outcomes (greater preservation of FFM and greater reduction of body mass, respectively) [25].

All statistical analyses and visualizations were performed in R (version 4.1.1; The R Foundation for Statistical Computing).

## 3 Results

### 3.1 Included Studies

**FIGURE 1:**
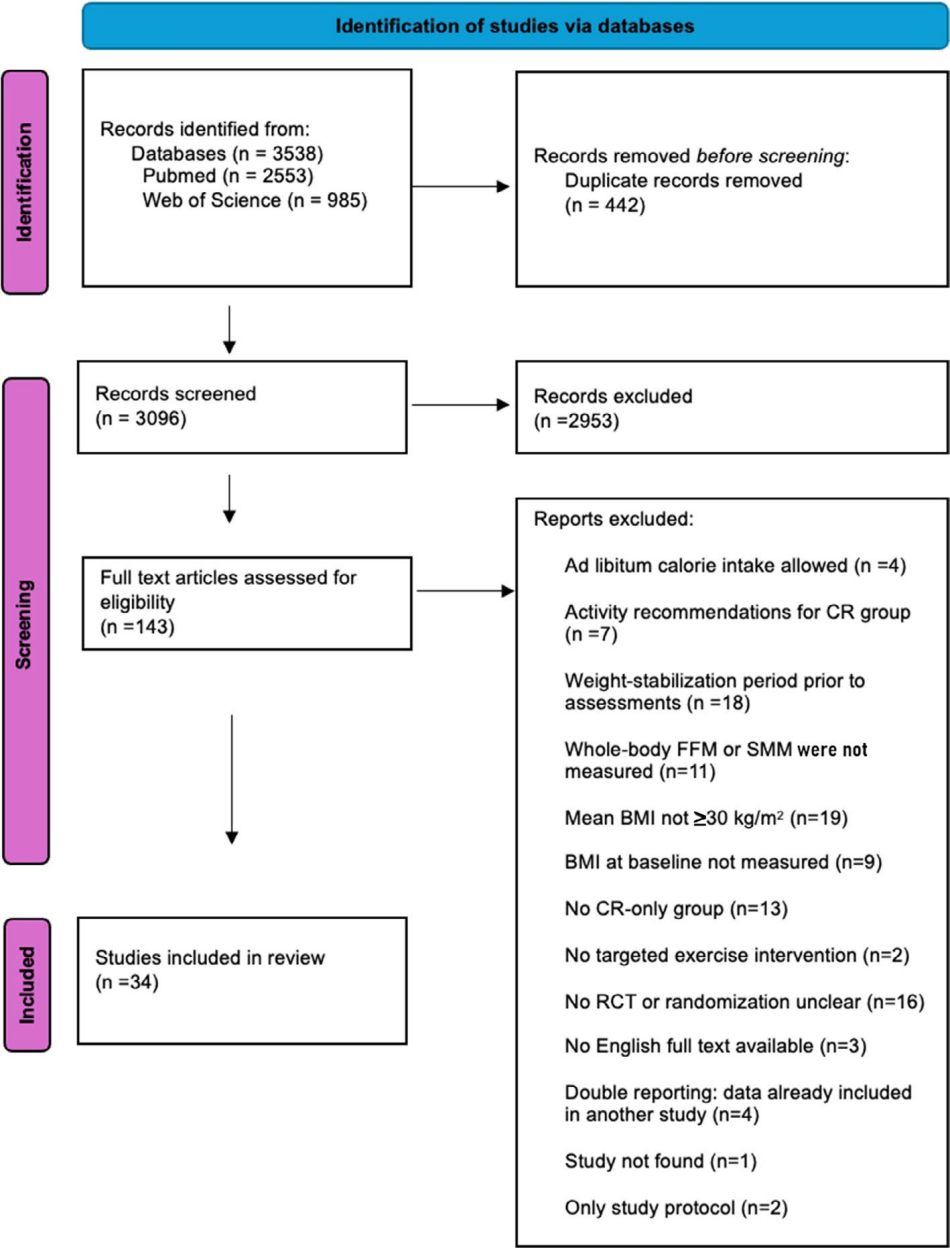
PRISMA flow chart.

The search initially yielded 3538 records. After removal of duplicates, 3096 unique citations were screened by title and abstract. Of these, 2953 records were excluded for not meeting the predefined eligibility criteria. A total of 143 articles underwent full‐text assessment, with 109 subsequently excluded due to inappropriate study design, missing or insufficient outcome data, or other reasons. In total, 34 studies met all inclusion criteria and were included in the meta‐analysis (Figure 1).

### 3.2 Study Characteristics

The included studies comprised a total of 1455 participants, with sample sizes ranging from 10 to 100 participants (based only on participants included in the present meta‐analysis). Two studies included only men [26, 27], 15 included only women [28, 29, 30, 31, 32, 33, 34, 35, 36, 37, 38, 39, 40, 41, 42], and 17 studies included participants of both sexes [43, 44, 45, 46, 47, 48, 49, 50, 51, 52, 53, 54, 55, 56, 57, 58, 59]. Mean participant age ranged from 29 to 70 years. None of the included studies used pharmacological agents (e.g., incretin mimetics) for weight loss. CR diets and exercise training interventions varied across studies. Detailed descriptions of participants, diet and exercise protocols are provided in File S2.

### 3.3 Quality of Randomized Controlled Trials

The methodological quality of the included studies, assessed using the PEDro scale, ranged from 4 to 7, with an average score of 5 (indicating “fair” quality). Using the RoB2 tool, 59% of studies (*n* = 20) were rated as having some concerns, and 41% (*n* = 14) as having a high risk of bias. Detailed scoring results are provided in Files S3 and S4.

### 3.4 CR With and Without Exercise Training on FFM: Meta‐Analytic Data

**FIGURE 2:**
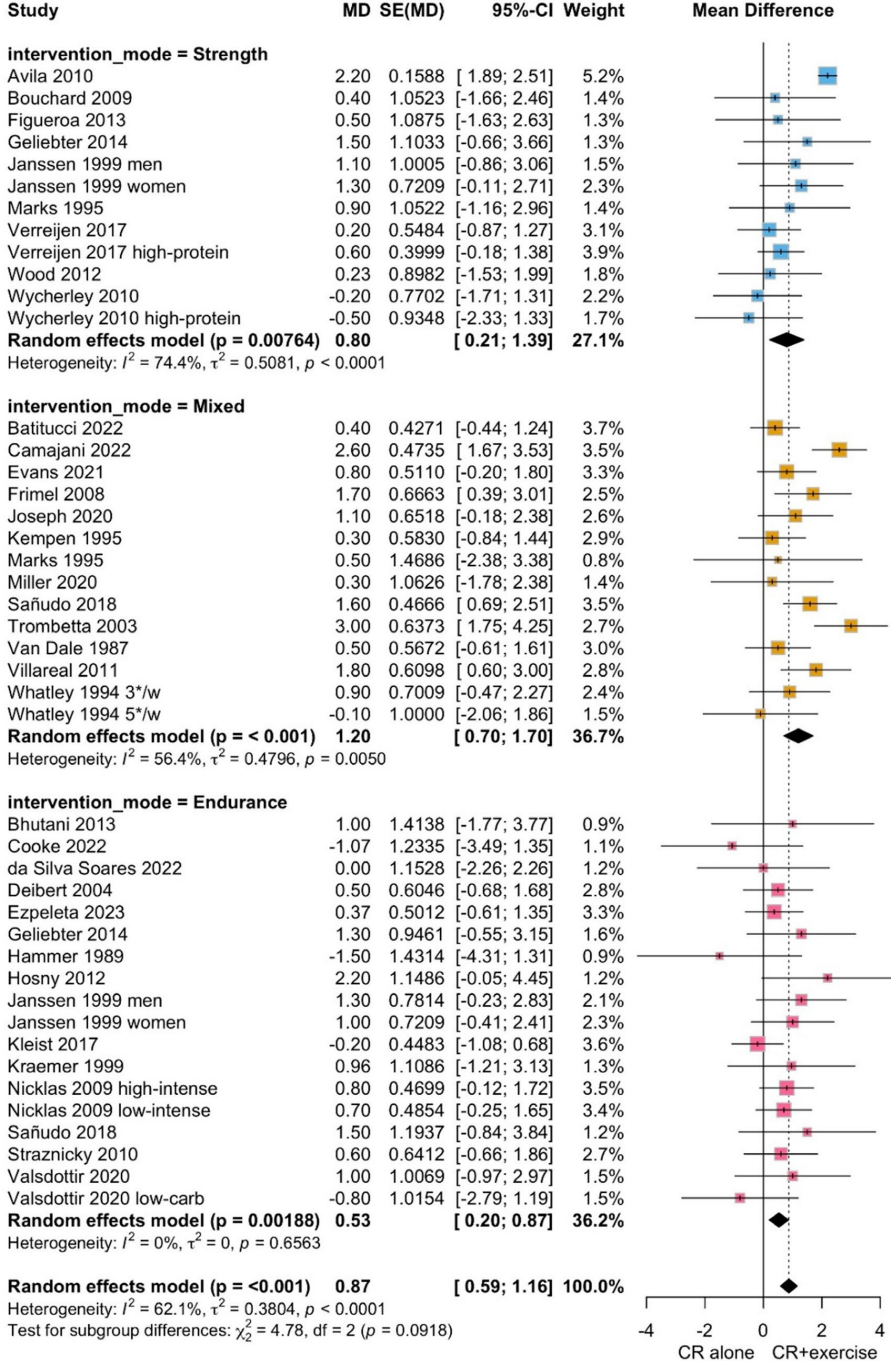
Forest plot of the pooled effect of calorie restriction combined with exercise training (CR + exercise) versus CR alone on fat‐free mass (FFM), expressed as mean differences with 95% confidence intervals. Studies are grouped by mode of training (strength, mixed, endurance) and weighted by inverse variance. Squares represent individual study effects and diamonds represent subgroup and overall pooled estimates from random‐effects models. Positive values on the x‐axis indicate higher FFM in the CR + exercise group. Colours denote training mode of subgroups. The figure also presents heterogeneity within each subgroup and overall heterogeneity, as well as the test for subgroup differences.

Across all included studies, CR combined with exercise training showed a significant positive effect on FFM compared with CR alone (*p* < 0.001), with the pooled estimate favouring CR combined with exercise training (MD = +0.87 kg, Figure 2). Overall heterogeneity was moderate (I^2^ = 62.1%, *p* < 0.001).

Subgroup analyses showed no significant differences between modes of training (*p* = 0.092; Figure 2). However, the effects were larger for resistance training alone (MD = +0.80 kg) and for combined resistance and endurance training (MD = +1.20 kg) than for endurance training alone (MD = 0.53 kg), suggesting that inclusion of resistance training may yield more favourable FFM outcomes. When stratified by sex, pooled effects were similar across subgroups, with no evidence of subgroup differences (*p* = 0.732).

For FFM, the leave‐one‐out analysis indicated that the pooled effect was stable when each study was removed sequentially. Across iterations, the re‐estimated pooled effects remained close to the original overall estimate (MD = +0.87 kg) and showed no meaningful change in direction. The pooled estimate ranged from +0.81 kg (minimum) to +0.92 kg (maximum), indicating that no single study disproportionately influenced the overall findings.

Twelve studies required SD change replacement for FFM. The pooled effect changed trivially from MD = +0.87 kg to MD = +0.81 kg and remained statistically significant (*p* < 0.001) when these studies were excluded from analysis.

Meta regression indicated no significant association between the total number of training sessions and the intervention effect (β = −0.006, SE = 0.007, *p* = 0.384; File S5; Figure S1A). The moderator did not explain the between‐study heterogeneity (*R* ^2^ = 0.7%), indicating that variation in the total number of sessions did not account for differences in effect sizes. Residual heterogeneity remained moderate (I^2^ = 50.3%).

Additional meta regression analyses were conducted to examine whether the magnitude of body mass loss moderated the effect of exercise on FFM. No significant moderating effect was observed for body mass change in the control arm (β = −0.003, *p* = 0.945), in the intervention arm (β = 0.011, *p* = 0.762), or the between‐group MD in body mass (β = 0.070, *p* = 0.403). In all three models, the proportion of heterogeneity explained by the moderator was 0%, indicating that the beneficial effect of exercise on FFM was not materially explained by the magnitude of weight loss. Residual heterogeneity remained moderate across models, with I^2^ values ranging from approximately 50% to 52%.

### 3.5 CR With and Without Exercise Training on SMM

Only two studies reported outcomes for SMM. Reljic et al. [57] observed a mean SMM reduction of −0.7 kg following CR alone, compared with −0.4 kg with CR combined with 1 set of resistance training, and −0.2 kg with CR combined with three sets of resistance training in a mixed‐sex cohort. Additionally, one intervention group performing 2 sets of whole‐body electromyostimulation training (WB‐EMS) showed a mean reduction of −0.9 kg. Although there was no significant time x group effect (analysis of variance), only the control and WB‐EMS groups showed significant muscle mass losses post‐compared to pre‐intervention. Janssen et al. [59] also reported that adding exercise attenuated SMM loss during CR. Statistical analyses revealed significant treatment effects for pre‐ to post‐intervention change scores adjusted for baseline values (analysis of covariance). In men, CR alone resulted in a mean SMM loss of −2.6 kg compared with −0.9 kg for CR combined with endurance training, and −1.0 kg for CR combined with resistance training. In women, CR alone resulted in a mean SMM loss of −1.2 kg compared with −0.5 kg for CR combined with endurance training, and −0.7 kg for CR combined with resistance training.

### 3.6 CR With and Without Exercise Training on Total Body Mass: Meta‐Analytic Data

**FIGURE 3:**
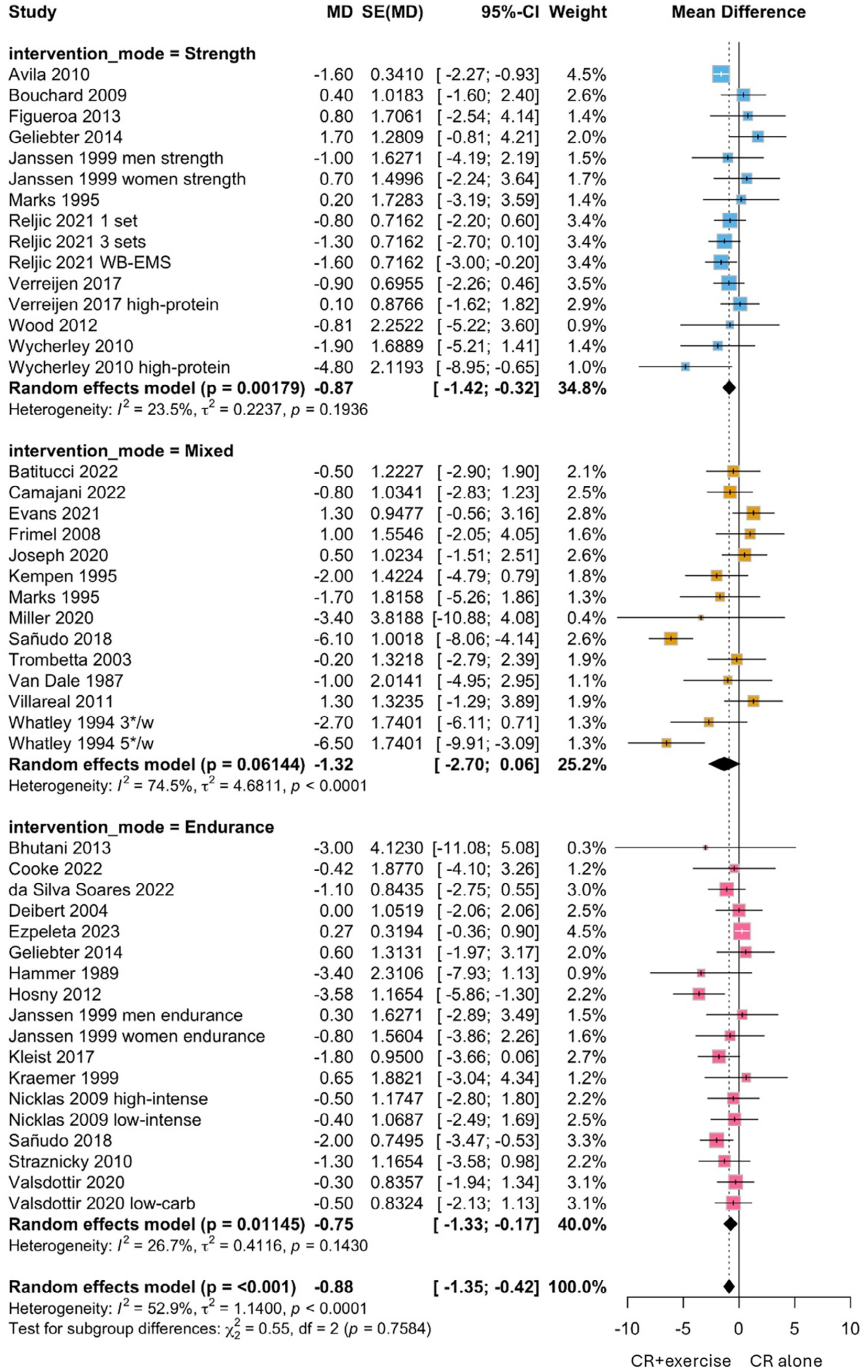
Forest plot of the pooled effect of calorie restriction combined with exercise training (CR + exercise) versus CR alone on body mass, expressed as mean differences with 95% confidence intervals. Studies are grouped by mode of training (strength, mixed, endurance) and weighted by inverse variance. Squares represent individual study effects, and diamonds represent subgroup‐specific and overall pooled estimates from random‐effects models. Negative values on the x‐axis indicate greater reductions in body mass, reflecting the desired direction of effect for weight loss. Colours denote training mode of subgroups. The figure also presents heterogeneity within each subgroup and overall heterogeneity, as well as the test for subgroup differences.

Across all included studies, CR combined with exercise training had a significant effect on body mass compared with CR alone, with the pooled estimate favouring CR combined with exercise training (MD = −0.88 kg, *p* < 0.001; Figure 3). Negative MD values indicate greater reductions in body mass, reflecting the desired direction of effect for weight loss. Overall heterogeneity was moderate (I^2^ = 52.9%, *p* < 0.001).

Subgroup analyses by mode of training showed no significant differences between modalities (*p* = 0.758; Figure 3). When stratified by sex, pooled effects were similar across subgroups, with no evidence of subgroup differences (*p* = 0.695).

The leave‐one‐out analysis indicated that the pooled effect remained stable when each study was removed sequentially. Across iterations, the re‐estimated pooled effects remained close to the original overall estimate (MD = −0.88 kg) and showed no meaningful change in direction. The pooled estimate ranged from −0.94 kg (minimum) to −0.73 kg (maximum), indicating that no single study disproportionately influenced the overall finding for body mass.

Twelve studies required SD change replacement for body mass. The pooled effect changed trivially from MD = −0.88 kg to MD = −0.72 kg and remained statistically significant (*p* < 0.001) when these studies were excluded from analysis.

In the meta‐regression analysis (File S5; Figure S1B), the total number of exercise training sessions was not significantly associated with effects on body mass (β = 0.015, SE = 0.013, *p* = 0.225). The moderator explained a small proportion of between‐study heterogeneity (R^2^ = 13.7%), and residual heterogeneity remained moderate (I^2^ = 49.7%).

### 3.7 CR With and Without Exercise Training on FFM and Total Body Mass: Illustrative Data

**FIGURE 4:**
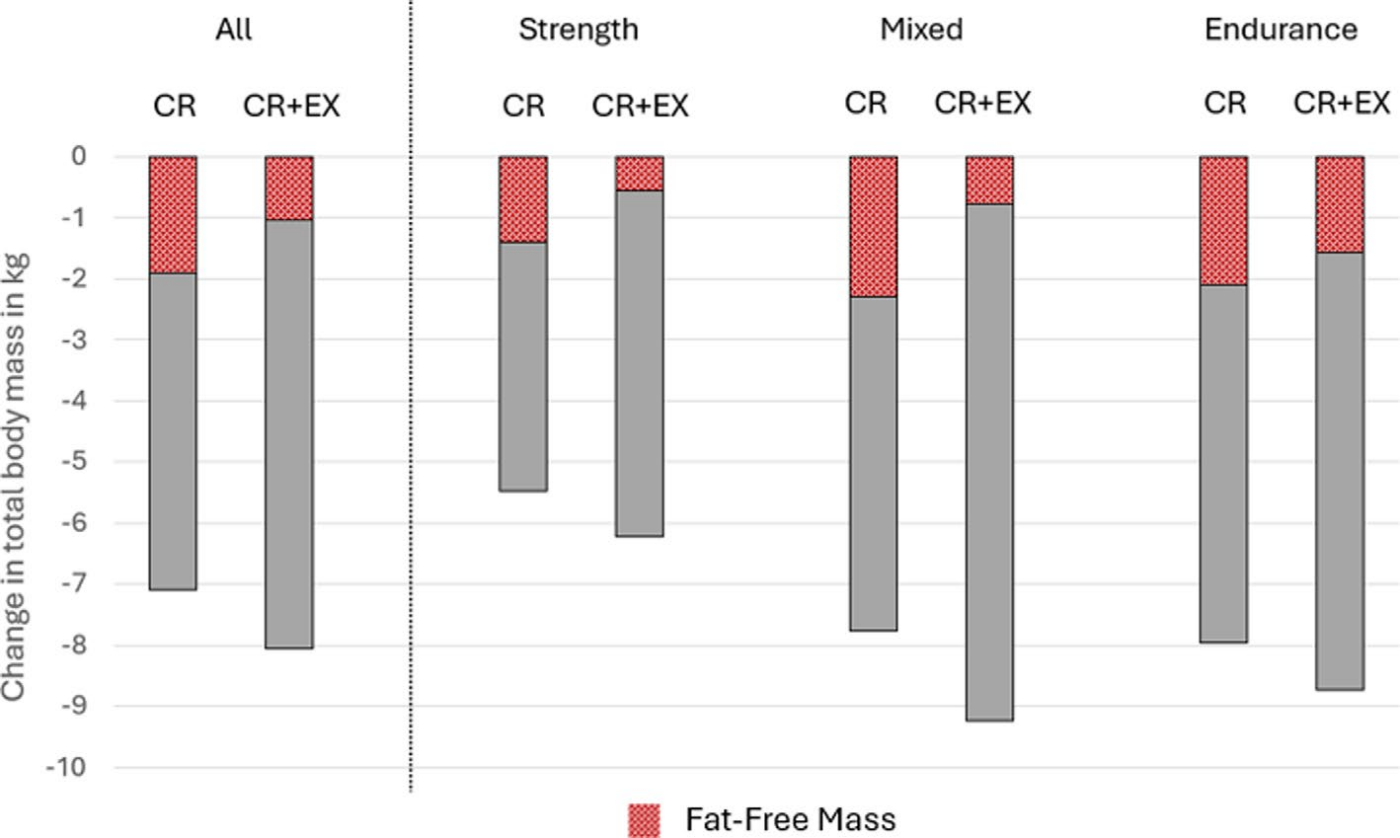
Illustration of the effects of calorie restriction (CR) alone and CR combined with exercise training on changes in fat‐free mass (FFM) and total body mass. Data are presented as weighted means.

For illustrative purposes, weighted mean changes in FFM and total body mass were calculated from all study arms reporting both outcomes. The data are presented in Figure 4. On average, exercise training reduced FFM loss during CR by nearly half (all data: 45.7%, strength training: 60.6%, mixed training: 66.4%, endurance training: 25.8%).

When examining individual study arms, FFM was fully preserved or even increased in 10 of 44 exercise training interventions during CR (across 8 of 34 studies), whereas a clear FFM loss was observed in the CR‐only groups [33, 35, 39, 43, 47, 50, 52, 53].

### 3.8 Publication Bias

For FFM, visual inspection of the funnel plot suggested asymmetry (File S6: Figure S2A). This was confirmed by Egger's regression test, which indicated statistically significant funnel plot asymmetry (t in negative direction, absolute value 4.44, df = 42, *p* < 0.001). The bias estimate was also negative, with an absolute value of 1.77 (SE = 0.399). Together, these findings suggest small‐study effects, and potential publication bias should be considered when interpreting the pooled effect.

For body mass, visual inspection of the funnel plot did not suggest pronounced asymmetry (File S6: Figure S2B). Consistent with this, Egger's regression test showed no evidence of funnel plot asymmetry (t = −0.76, df = 45, *p* = 0.452), with a bias estimate of −0.32 (SE = 0.419). Overall, these findings indicate no clear evidence of small‐study effects.

### 3.9 Network Meta‐Analysis: FFM and Body Mass

**FIGURE 5:**
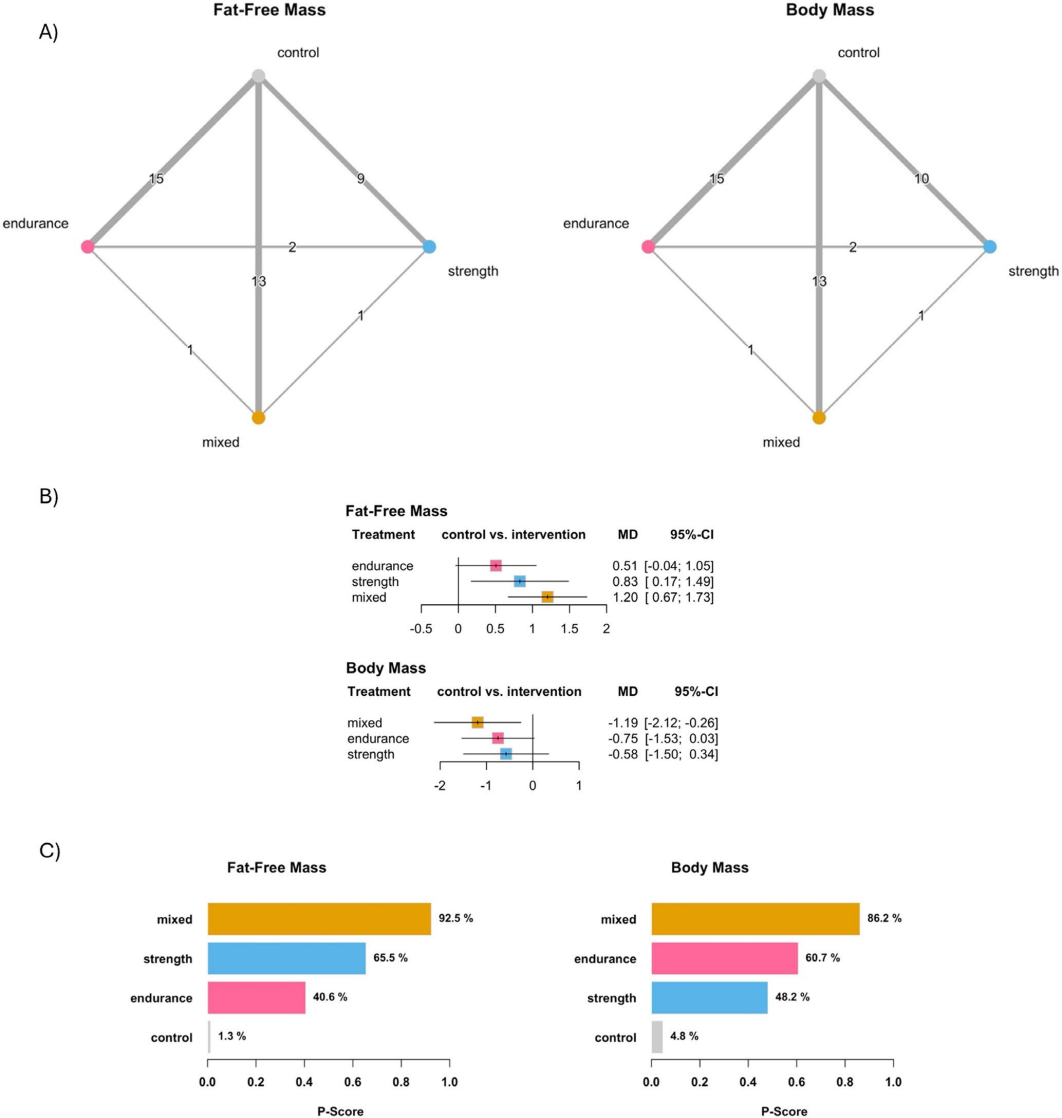
Network meta‐analyses. (A) Network plots of eligible interventions for fat‐free mass (FFM) and body mass. Nodes represent CR alone (labelled “control”), endurance, strength and mixed training. Line thickness reflects the number of direct comparisons contributing to each edge. (B) Forest plots of the network meta‐analyses comparing exercise modalities with CR alone for FFM and body mass. Effects are reported as mean differences with 95% confidence intervals. (C) P‐score rankings for FFM and body mass. Higher P‐scores indicate more favourable outcomes.

The network meta‐analysis for FFM included 33 studies that contributed 41 pairwise comparisons across four treatment nodes (CR alone as the reference, endurance, strength and mixed; Figure 5A). Compared with CR alone, mixed training and strength training significantly preserved FFM, whereas endurance training fell just short of statistical significance. Mixed training showed the largest effect (MD = +1.20 kg, 95% CI: +0.67, +1.73; *p* < 0.001), followed by strength training (MD = +0.83 kg, 95% CI: +0.17, +1.49; *p* = 0.013), and endurance training (MD = +0.51 kg, 95% CI: −0.04, +1.05; *p* = 0.067; Figure 5B). Heterogeneity within designs was moderate (I^2^ = 60.5%), and inconsistency between designs was not statistically significant (Q = 6.56, df = 6, *p* = 0.363). The P‐score ranking confirmed mixed training as the most effective intervention for preserving FFM, followed by strength and endurance training, while CR alone ranked lowest (Figure 5C).

For body mass, the network included 34 studies that contributed 42 pairwise comparisons across the four treatment nodes (Figure 5A). Mixed training produced a significantly greater reduction in body mass than CR alone (MD = −1.19 kg, 95% CI: −2.12, −0.26; *p* = 0.012), while endurance training narrowly missed statistical significance (MD = −0.75 kg, 95% CI: −1.53, +0.03; *p* = 0.059). Strength training did not differ significantly from CR alone (MD = −0.58 kg, 95% CI: −1.50, +0.34; *p* = 0.219; Figure 5B). Heterogeneity was moderate (I^2^ = 57.4%) and inconsistency between designs was substantial (Q = 35.52, df = 6, *p* < 0.001), indicating that direct and indirect evidence did not agree uniformly across designs. The P‐score ranking placed mixed training at the top, followed by endurance training, while strength training and CR alone ranked lowest in terms of P‐scores (Figure 5C).

## 4 Discussion

Across the included trials, the present pairwise meta‐analysis shows that adding exercise training to a CR diet results in a significant improvement in FFM compared with CR alone. Although subgroup analyses did not reveal significant differences between modes of training, point estimates suggested larger benefits when strength training is included in the intervention. The network findings closely converged with the results of the pairwise meta‐analysis. Mixed and strength training preserved FFM significantly more effectively than CR alone, whereas endurance training narrowly missed statistical significance. Mixed training emerged as the strategy with the greatest potential for preserving FFM in both analyses. The absence of significant inconsistency between direct and indirect evidence in the FFM network strengthens confidence in the robustness of this result [24].

For body mass, the pairwise meta‐analysis and the network analysis showed more pronounced divergence. Whereas the pairwise subgroup analysis found no significant differences between training modalities, the network analysis identified mixed training as the only modality that showed a significantly greater reduction in body mass compared with CR alone, while endurance training narrowly missed statistical significance (*p* = 0.059) and strength training did not differ from CR alone (*p* = 0.219).

However, the body mass network showed substantial inconsistency that is a formal signal that direct and indirect comparisons did not yield uniform effect estimates across the included studies and is the most plausible explanation for the discrepancy observed between the two analytical frameworks [24]. Potential sources of this inconsistency include differences in intervention duration, magnitude of the prescribed calorie deficit, exercise volume and adherence across the trials that contributed to each contrast. These factors are unlikely to be fully balanced across direct and indirect comparisons. In the presence of such inconsistency, the ranking obtained from the body mass network should be interpreted with caution, particularly where direct evidence for a specific contrast is sparse. For body mass, the divergent findings and the inconsistency detected in the network suggest that the optimal modality depends on the structure of the intervention and on the specific trial context.

Our findings are broadly in line with a previous meta‐analysis conducted by Sardeli et al. [13] who reported no differences in reductions of total body mass and fat mass between CR combined with strength training and CR alone, while FFM loss was 93.5% lower for CR combined with strength training. Data from our pairwise meta‐analysis show that FFM loss was attenuated by 54%, indicating a smaller but still clinically meaningful protective effect. Several factors may account for this discrepancy. Sardeli et al. [13] included exclusively older adults, a population group characterized by age‐related muscle loss that may be further exacerbated under CR [60]. Strength training is a primary stimulus for protein synthesis, and changes in muscle mass may be more pronounced in untrained individuals with lower muscle mass [61]. The larger number of included trials in our analysis (33 vs. 6) improves generalizability but introduces greater heterogeneity in participant characteristics and intervention protocols. Moreover, Sardeli's analysis focused exclusively on strength training, whereas our study included strength, endurance and mixed training interventions.

Based on our analyses, mixed training may offer particular potential for preserving FFM, likely due to the substantial mechanical and metabolic stimuli it imposes on skeletal muscle [62]. This dual stimulus may be particularly protective under conditions of energy deficit, where substrate availability is limited and anabolic signalling is attenuated.

Overall, maintaining or increasing FFM during CR remains inherently challenging. Meta‐analytic evidence by Murphy & Koehler [63] suggests that meaningful increases in FFM are difficult to achieve through strength training under conditions of energy deficit, as reduced energy availability may constrain anabolic adaptations. In our analysis, however, higher calorie deficits were not consistently associated with greater FFM reductions, suggesting that the magnitude of energy restriction alone is not the only determinant and that other factors, such as habitual daily physical activity outside structured training programmes may also play an important role [64].

The relevance of the specific method of CR and patterns of food intake warrants further investigation. It remains unclear whether time‐restricted eating approaches offer benefits for skeletal muscle energy and protein metabolism, and for FFM preservation during CR. Four studies in our dataset implemented intermittent fasting protocols, such as alternate‐day fasting [28, 45, 50, 55]. In these studies, FFM loss ranged between −1.2 and + 0.4 kg and were below the weighted mean FFM loss observed across all included studies (−1.9 kg). However, Keenan et al. [65] compared a 5:2 intermittent fasting approach with continuous energy restriction (with matched energy and protein intake), each combined with strength training, and reported similar changes in total FFM between groups, with no clear advantage of intermittent fasting over continuous CR.

Approximately half of the studies included in our meta‐analysis implemented a diet with increased protein content. Higher protein intake, particularly when combined with strength training, is generally considered beneficial for attenuating FFM loss during energy restriction [66]. However, the health implications of increased protein intake remain controversially debated, especially in individuals with pre‐existing medical conditions, despite the limited robust evidence of potentially adverse effects [67].

When exploring potential sources of effect heterogeneity, several studies emerged as negative outliers, showing less favourable outcomes when CR was combined with exercise. Two of these studies employed high‐intensity interval training (HIIT) protocols [31, 55]. One possible explanation is that the training loads may have been too high, and that excessive physical exertion without sufficient recovery time may have negatively affected anabolic processes [68]. However, Sañudo et al. [52] used a comparable HIIT protocol, and reported effects in the desired direction. Future research is therefore needed.

Furthermore, the additional meta regression analyses did not support the assumption that the magnitude of weight loss is a major determinant of the FFM preserving effect of exercise. Instead, the beneficial effect of exercise appeared relatively robust across different degrees of body mass reduction, although relevant residual heterogeneity remained unexplained.

Supervision may also have influenced the observed effects. Among the 34 included trials, five implemented one‐to‐one personal training [26, 27, 43, 47, 57], with some reporting particularly pronounced positive effects on FFM changes [43, 47]. While the majority of included trials incorporated some form of supervised exercise training (predominantly in group‐based supervised settings), existing evidence suggests that supervised strength training generally yields superior adaptive outcomes compared with unsupervised programmes [69].

While our pairwise meta‐analysis focused on the effects of CR with or without exercise training, we had initially intended to examine potential interactions with incretin mimetics. However, evidence on FFM preservation during treatment with incretin mimetics in combination with exercise training remains limited, and no RCTs met the inclusion criteria for our meta‐analysis. A recent case series involving treatment with semaglutide or tirzepatide reported that structured exercise training (including strength training at least 3 x/week), combined with adequate protein intake, enabled even increases in lean soft tissue despite substantial reductions in weight and fat mass in two of three patients [70]. These findings suggest that integrating exercise training with appropriate nutritional strategies may help mitigate or fully prevent FFM loss during pharmacologically‐induced weight reduction. Nevertheless, RCTs are needed before definitive conclusions can be drawn.

In addition to changes in FFM, changes in total body weight were also analysed. We observed that adding exercise training to CR resulted in additional weight loss, likely driven by additional calorie consumption due to exercise training [71]. It can generally be assumed that higher training volumes increase total calorie consumption and result in more substantial changes in body mass (particularly loss of fat mass). This is supported by Whatley et al. [36] who observed larger decreases in body mass with five training sessions per week compared with only three sessions. Furthermore, exercise training may have a positive effect on basal metabolic rate, which typically decreases during CR [72]. In this context, it is also worth noting that exercise training may counteract the downregulation of energetically relevant components of the mitochondrial respiratory chain in skeletal muscle observed during CR [73, 74].

### 4.1 Strength and Limitations

The primary strengths of this study include the exclusive inclusion of studies with direct head‐to‐head comparisons (CR alone and CR + exercise training) in the pairwise meta‐analysis as well as the availability of additional evidence provided by the network meta‐analysis. However, several limitations must be mentioned. The included studies varied considerably in terms of exercise interventions (type of exercise, duration of individual training sessions, training frequency or intervention duration, presence or absence of warm‐up/cool‐down routines) and dietary protocols (magnitude of calorie deficit, dietary composition and macronutrient ratio), contributing to substantial heterogeneity among the included studies. It is noteworthy that heterogeneity was only observed in strength training and mixed training interventions, but not in endurance training. This pattern may reflect greater variability in the design of strength training protocols across the studies, which could explain differences in results. Furthermore, several influencing factors, such as medication use and the presence of chronic diseases, were not accounted for in the present analyses. In addition, FFM and muscle mass were measured using diverse measurement techniques, including dual‐energy x‐ray absorptiometry (DXA) (*n* = 14 studies), bioelectrical impedance analysis (BIA) (*n* = 9 studies), air displacement plethysmography (*n* = 4 studies), magnetic resonance imaging (MRI) (*n* = 1 study), and hydrodensitometry (*n* = 6 studies). Each method relies on distinct physiological and compartmental assumptions and varies in precision, modelling approach, and sensitivity to biological variability [75]. Such differences may introduce bias, as differences in validity and accuracy across body composition assessment methods can influence FFM estimates, particularly during weight loss, where shifts in body water distribution and changes in non‐muscle lean tissue may affect FFM independently of actual skeletal muscle mass [76]. Findings for FFM should also be interpreted with caution, as evidence of publication bias (funnel plot asymmetry and a significant Egger's test) suggests that the pooled effect from the pairwise meta‐analysis may be overestimated. In addition, according to RoB2 ratings, deviations from the intended interventions often represent a high risk of bias in the included studies.

### 4.2 Conclusions

In conclusion, this up‐to‐date pairwise meta‐analysis demonstrates that exercise training can effectively help preserve FFM during CR in individuals who are overweight/obese. On average, nearly half of FFM loss was prevented by EX. However, these results should be interpreted with caution, as evidence of publication bias suggests that the pooled effect may be overestimated. Findings from the network analysis further indicate that mixed and strength training interventions may yield larger effects on FFM preservation. Data on SMM are limited and do not permit definitive conclusions. The present study provides valuable new insights for optimizing weight loss strategies.

## Funding

The authors have nothing to report.

## Conflicts of Interest

C.B. received lecture/consultation fees from Abbott, Lilly, and Novo Nordisk. All other authors declare no conflicts of interest.

## Supporting information


**File S1:** Full search string “(calorie restriction OR energy restriction OR diet‐induced weight loss OR calorie deficit OR weight loss diet OR GLP‐1 OR GLP1 OR glucagon‐like peptide OR glucagon like peptide OR GIP OR glucose‐dependent insulinotropic peptide OR Semaglutid OR Liraglutid OR Albiglutid OR Dulaglutid OR Exenatid OR Lixisenatid OR Tirzepatid) AND (exercise OR physical Activity OR training OR resistance OR strength OR endurance) AND (muscle mass OR fat‐free mass OR fat free mass OR lean mass OR lean body mass OR lean‐body mass OR skeletal muscle OR MM OR FFM OR LM) AND (obes* OR BMI > 30 OR overweight OR adipos*).”
**File S2:** Study characteristics.
**File S3:** PEDro scoring details.
**File S4:** RoB2 scoring details.
**File S5:** Meta regression analyses.
**Figure S1:** Meta regression of the intervention effect as a function of the total number of exercise training sessions. Panel A presents results for fat‐free mass (FFM), and Panel B presents results for body mass. Points represent individual study effects weighted by total sample size, with colours indicating mode of training. Solid lines denote fitted meta regression slopes, and shaded areas represent 95% confidence bands. Each panel reports the regression coefficient, standard error, *p*‐value, and the proportion of heterogeneity explained (R^2^).
**File S6:** Funnel plots.
**Figure S2:** Funnel plots for the meta‐analyses of fat‐free mass (FFM) (A) and body mass (B). Each point represents an individual study effect plotted against its standard error, with the vertical line indicating the pooled random effects estimate and diagonal lines representing the expected pseudo confidence limits. Colours denote mode of training (orange = strength, blue = mixed, red = endurance).

## Data Availability

These meta‐analyses exclusively used data extracted from publicly available RCTs. All data are accessible in the published articles cited in the manuscript.
