# Supplementary material for: Effects of Calorie Restriction With and Without Strength, Endurance or Mixed Training on Fat‐Free and Skeletal Muscle Mass in Overweight or Obese Individuals—A Systematic Review With Pairwise Meta‐Analysis and Network Meta‐Analysis of Randomized Controlled Studies
Source: Diabetes Obes Metab. 2026 May 17;28(8):6810–23. doi: 10.1111/dom.70873 (PMC13341414; doi:10.1111/dom.70873)
Supplement: Supplementary file 1 — File S1: Full search string “(calorie restriction OR energy restriction OR diet‐induced weight loss OR calorie deficit OR weight loss diet OR GLP‐1 OR GLP1 OR glucagon‐like peptide OR glucagon like peptide OR GIP OR glucose‐dependent insulinotropic peptide OR Semaglutid OR Liraglutid OR Albiglutid OR Dulaglutid OR Exenatid OR Lixisenatid OR Tirzepatid) AND (exercise OR physical Activity OR training OR resistance OR strength OR endurance) AND (muscle mass OR fat‐free mass OR fat free mass OR lean mass OR lean body mass OR lean‐body mass OR skeletal muscle OR MM OR FFM OR LM) AND (obes* OR BMI > 30 OR overweight OR adipos*).” File S2: Study characteristics. File S3: PEDro scoring details. File S4: RoB2 scoring details. File S5: Meta regression analyses. Figure S1: Meta regression of the intervention effect as a function of the total number of exercise training sessions. Panel A presents results for fat‐free mass (FFM), and Panel B presents results for body mass. Points represent individual study effects weighted by total sample size, with colours indicating mode of training. Solid lines denote fitted meta regression slopes, and shaded areas represent 95% confidence bands. Each panel reports the regression coefficient, standard error, p‐value, and the proportion of heterogeneity explained (R2). File S6: Funnel plots. Figure S2: Funnel plots for the meta‐analyses of fat‐free mass (FFM) (A) and body mass (B). Each point represents an individual study effect plotted against its standard error, with the vertical line indicating the pooled random effects estimate and diagonal lines representing the expected pseudo confidence limits. Colours denote mode of training (orange = strength, blue = mixed, red = endurance). [file DOM-28-6810-s001.docx]

**Supplemental data file 1**. Full search string

“(calorie restriction OR energy restriction OR diet-induced weight loss OR calorie deficit OR weight loss diet OR GLP-1 OR GLP1 OR glucagon-like peptide OR glucagon like peptide OR GIP OR glucose-dependent insulinotropic peptide OR Semaglutid OR Liraglutid OR Albiglutid OR Dulaglutid OR Exenatid OR Lixisenatid OR Tirzepatid) AND (exercise OR physical Activity OR training OR resistance OR strength OR endurance) AND (muscle mass OR fat-free mass OR fat free mass OR lean mass OR lean body mass OR lean-body mass OR skeletal muscle OR MM OR FFM OR LM) AND (obes* OR BMI>30 OR overweight OR adipos*)”

**Supplemental data file 2.** Study characteristics

| **Study** | **Country** | **Duration (months)** | **Total number of partici-pants (of all study groups) at base-line** | **Total number of partici-pants included in the final analysis** | **Mean age (±SD) of analyzed partici-pants (unless otherwise stated)** | **Sex** | **Structured exercise programme**  **(strength, endurance or mixed and additional information if available)** | **Dietary intervention**  **(available information)** | **Per-protocol (PP) or intention-to-treat (ITT) analysis** | **Body composition analysis technique** |
| --- | --- | --- | --- | --- | --- | --- | --- | --- | --- | --- |
| Avila  2010 | USA | 2.5 | 27 | Diet:  11  Diet+  exercise: 15 | Diet:  67±5  Diet+  exercise:  66±4 | Males and females | Strength:  3*/w, 4 sets, 8-12 reps, 6 exercises, supervised | 10% energy restriction, dietary  goal: 5% weight loss | PP | Air displacement plethysmo-graphy |
| Batitucci  2022 | Brazil | 2 | 60 | Diet:  15  Diet+  exercise: 10 | Diet:  30±5  Diet+  exercise:  32±4 | Females | Mixed:  3*/w, high-intensity interval training, 18 min at 70-85% HRmax (multi-stimulating conditioning circuit training), supervised | 2:5 intermittent fasting: 2 d/w, 25% total daily energy expenditure (~600 kcal; 6:18 h), 5 days ad libitum | PP | Air displacement plethysmo-graphy |
| Bhutani  2013 | USA | 3 | 83 | Diet:  25  Diet+  exercise: 18 | Diet:  42±2  Diet+  exercise:  45±5 | Males and females | Endurance:  3*/w, 25-40 min cycling & elliptical machines, 60-75% HRmax, supervised | Alternate day fasting: 25% total daily energy expenditure on fasting days, ad libitum on feeding days | ITT | Bioelectrical Impedance Analysis (BIA) |
| Bouchard 2009 | Canada | 3 | 48 | Diet:  11  Diet+  exercise: 12 | Diet:  61±5  Diet+  exercise:  64±5 | Females | Strength:  3*/w, 3 sets, 8 reps, 80% 1-RM, 9 exercises, supervised | Dietary goal: reducing body weight by 0.5-1.0 kg/w  (55C/15P/30F) | PP | Dual-energy  X-ray absorptiometry (DXA) |
| Camajani 2022 | Italy | 1.5 | 24 | Diet:  12  Diet+  exercise: 12 | Diet:  56±7  Diet+  exercise:  57±4 | Males and females | Mixed:  2*/w, 30-35 min interval training with functional exercises, 20 s exercise/10 s rest, supervised (remote) | 780-800 kcal/d,  Low-carbohydrate diet (13.5C/46.1P/40.4F) | PP | BIA |
| Cooke  2022 | Australia | 4 | 45 | Diet:  12  Diet+  exercise: 11 | Diet:  37±6  Diet+  exercise:  39±7 | Males and females | Endurance:  3*/w, cycling intervals with 4-6*20 s workout at 150% VO_2peak_, 40 s active rest, supervised | 2:5 intermittent fasting:  2 d/w fasting with 500-600 kcal/d  5 d/w ad libitum calorie intake | ITT | DXA |
| Da Silva Soares  2022 | Brazil | 3 | 39 | Diet:  13  Diet+  exercise: 13 | Diet:  60±8  Diet+  exercise:  60±7 | Males and females | Endurance:  3*/w, running 1*12 min high-intensity interval exercise & 1*12 min continuous moderate-intense training, >60% HRmax for 70% of total training time, supervised | 500 kcal/d energy restriction | PP | DXA |
| Deibert  2004 | Germany | 6 | 90 | Diet:  28  Diet+  exercise: 27 | N/A | Males and females | Endurance:  2*/w, 60 min,  supervised | Weeks 1-6: 1000-1200 kcal/d, weeks 7-24:1500-1700 kcal/d, partly with soy-protein-yoghurt meal replacement | PP | Air displacement plethysmo-graphy |
| Evans  2021 | USA | 6 | 81 | Diet:  20  Diet+  exercise: 19 | Diet:  69±2  Diet+  exercise:  70±4 | Females | Mixed:  3*/w, 30 min endurance training at moderate intensity,  strength training with 2 sets, 8-10 reps, 65% 1-RM, exercises for major muscle groups,  supervised | 500 kcal/d energy restriction;  high-protein diet  (40C/30P/30F) | PP | DXA |
| Ezpeleta  2023 | USA | 3 | 80 | Diet:  20  Diet+  exercise: 20 | Diet:  44±16  Diet+  exercise:  44±13 | Males and females | Endurance:  5*/w, 60 min cycling, treadmill, elliptical machines, 65-80% HRmax, supervised | Alternate day fasting: 600 kcal on fasting days (dinner), ad libitum on feeding days | ITT | DXA |
| Figueroa  2013 | USA | 3 | 45 | Diet:  13  Diet+  exercise: 14 | Diet:  54±1 (SE)  Diet+  exercise:  54±1 (SE) | Females | Strength:  3*/w, 2-3 sets, 18-22 reps, 4 exercises, supervised | 1250kcal/d  (55-60C/20-25P, 20-25F) | PP | DXA |
| Frimel  2008 | USA | 6 | 30 | Diet:  15  Diet+  exercise: 15 | Diet:  70±5  Diet+  exercise:  69±4 | Males and females | Mixed:  3*/w, 30 min low-impact endurance training, strength training with 2-3 sets, 6-12 reps, 65-85% 1-RM, 9 exercises,  supervised | 750kcal/d energy restriction, dietary goal: 10% weight loss  (50C/20P/30F) | PP | DXA |
| Geliebter  2014 | USA | 2 | 81 | Diet:  17  Diet+  strength: 16  Diet+  endurance: 24 | Diet:  36±8  Diet+  strength:  35±6  Diet+  endurance:  36±8 | Males and females | Strength:  3*/w, 3 sets, reps to muscular failure, 8 exercises  Endurance:  3*/w, 8 min leg cycling + 8 min upper-body ergometer + 8 min leg cycling, 70% HRmax  Both supervised | ~1235kcal/d, 70% resting metabolic rate, formula diet/meal replacement  (24C/52P/24F) | PP | BIA |
| Hammer  1989 | USA | 4 | 36 | Diet:  8  Diet+  exercise:  6 | Diet:  34±6  Diet+  exercise:  33±8 | Females | Endurance:  5*/w, walking and/or jogging, 0.8-4.8 km, at 60-85% HRmax, supervised | 800kcal/d (63C/28P/9F) | PP | Hydrodensito-metry |
| Hosny  2012 | Egypt | 3 | 40 | Diet:  20  Diet+  exercise: 20 | Diet:  35±3  Diet+  exercise:  36±2 | Females | Endurance:  3*/w, 30 min treadmill walking, 70% HRmax,  supervised | 500-1000 kcal/d energy restriction  (55C/15P/30F) | PP | BIA |
| Jannsen  1999 | Canada | 4 | 71 | Diet (men): 10  Diet+  endurance (men):  10  Diet+  strength  (men):  10  Diet (women): 10  Diet+  endurance (women): 10  Diet+  strength (women): 10 | Diet (men): 46±2 (SE)  Diet+  endurance (men):  47±2 (SE)  Diet+  strength  (men):  38±4 (SE)  Diet (women):  40±2 (SE)  Diet+  endurance (women): 39±2 (SE)  Diet+  strength (women): 37±1 (SE) | Males and females | Strength:  3*/w, 1 set, 8-12 reps, 7 exercises  Endurance:  5*/w, 15-60 min walking, cycling, stair stepping, 50-85% HRmax  Both supervised | 1000 kcal/d energy restriction with <30% of energy from fat | PP | Magnetic resonance imaging (MRI) |
| Joseph  2020 | Israel | 2 | 145 | Diet:  21  Diet+  exercise: 41 | Diet:  56±6  Diet+  exercise:  53±7 | Females | Mixed:  3*/w with 1*/w endurance 1h, 1*/w strength 1h, (+1*/w stretching 1h), supervised? | 1000-1500 kcal/d, low-carbohydrate diet | PP | BIA |
| Kempen  1995 | Nether-lands | 2 | 20 | Diet:  10  Diet+  Exercise: 10 | Diet:  37±2 (SE)  Diet+  Exercise:  39±5 (SE) | Females | Mixed:  3*/w, 60-90 min dancing and fitness sessions (with strength and endurance training), 50-60% of VO_2max_,  partly supervised | Weeks 1-4: 480 kcal/d, weeks 5-8: 840kcal/d, partly formula diet/meal replacement | PP | Hydrodensito-metry |
| Kleist  2017 | Germany | 3 | 91 | Diet:  44  Diet+  exercise: 38 | 39±8  (no group-specific data) | Males and females | Endurance:  3*/w, 1h walking at 6 km/h, partly supervised | 500-800kcal/d energy restriction (42-45C/22-25P/32-35F) | PP | BIA |
| Kraemer  1999 | USA | 3 | 35 | Diet:  8  Diet+  exercise: 11 | Diet:  40±6  Diet+  exercise:  38±9 | Males | Endurance:  3*/w, 30-50 min treadmill walking, jogging, cycling, rowing, stair climbing, 70-80% of functional capacity,  supervised | 1500 kcal/d,  dietary goal: reducing body weight by 0.5-1.0 kg/w, partly formula diet/meal replacement | PP | Hydrodensito-metry |
| Marks  1995 | USA | 5 | 67 | Diet:  10  Diet+  strength: 11  Diet+  mixed:  9 | Diet:  38±8  Diet+  strength:  39±2  Diet+  mixed:  40±6 | Females | Strength:  3*/w, 2 sets, 12 reps, 7-8 exercises  Mixed:  3*/w, 1 set, 12 reps, 7-8 exercises and 12-24 min cycling, 70-85% HRmax  Both supervised | ~630 kcal/d energy restriction, low-fat diet | PP | Hydrodensito-metry |
| Miller  2020 | Australia | 12 | 60 | Diet:  26-28  Diet+  exercise: 28-29 | Diet:  36±10  Diet+  exercise:  38±8  (baseline data of 30 persons per group?) | Females | Mixed:  Up to 3*/w, 20-30 min endurance training/60-80% HR reserve and 30 min strength training, 1-3 sets, 8-10 reps, 6-10 exercises,  partly supervised  Supervised training sessions were supplemented with self-administered sessions | Weeks 1–12: 450–680 kcal/d, weeks 13–18: 800–880 kcal/d,  weeks 19–22: 1000–1400 kcal/d, weeks 23–52: 1200 kcal/d, partly formula diet/meal replacement | ITT | DXA |
| Nicklas  2009 | USA | 5 | 112 | Diet:  29  Diet+  low-intense exercise: 36  Diet+  high-intense exercise: 30 | Diet:  58±6  Diet+  low-  intense exercise:  58±6  Diet+  high-  intense exercise:  59±5  (baseline data of 34, 40 and 38 persons per group) | Females | Endurance low-intense:  3*/w, 20-55 min treadmill walking, 45-50% HR reserve  Endurance high-intense:  3*/w, 10-30 min treadmill walking, 70-75% HR reserve  Both supervised | 400kcal/d energy restriction  (50-60C/15-20P/25-30F) | PP | DXA |
| Reljic  2021 | Germany | 3 | 118 | Diet:  22  Diet+  strength  1 set:  23  Diet+  strength  3 sets:  23  Diet+  WB-EMS: 23 | Diet:  52±12  Diet+  strength  1 set:  54±12  Diet+  strength  3 sets: 54±11  Diet+  WB-EMS:  52±12  (baseline data of 30, 28, 29 and 31 persons per group) | Males and females | Strength 1 set:  2*/w, 1 set until muscular failure, 50-80% 1-RM, 5 exercises  Strength 3 sets:  2*/w, 3 sets until muscular failure, 50-80% 1-RM/5 exercises  Strength WB-EMS:  Bipolar impulses at a frequency of 85 Hz and a pulse width of  350 µs during 2 sets with 10 reps of light movements of the major muscle groups  All supervised | 500 kcal/d energy restriction with 1g protein/kg body weight/d | PP | BIA |
| Sañudo  2018 | Spain | 2 | 40 | Diet:  12  Diet+  endurance: 14  Diet+  mixed:  13 | Diet:  36±9  Diet+  endurance:  35±7  Diet+  mixed:  35±7  (baseline data of  13, 14 and 13 persons per group?) | Males and females | Endurance:  3*/w, 6-10 sets*1min of HIIT cycling at 90% HRmax  Mixed:  3*/w, 6-10 sets*1min of HIIT cycling at 90% HRmax + 6-10 sets of 1 min isometric squats on vibration platform 18-25Hz  Supervision? | 480kcal/d energy restriction  (50-55C/15P/30F) | PP | BIA |
| Straznicky 2010 | Australia | 3 | 64 | Diet:  20  Diet+  exercise: 20 | Diet:  55±1 (SE)  Diet+  exercise: 54±1 (SE) | Males and females | Endurance:  3-4*/w, 40 min cycling/65% HRmax, partly supervised | 600 kcal/d energy restriction (48C/22P/30F) | PP | DXA |
| Trombetta 2003 | Brazil | 4 | 59 | Diet:  24  Diet+  exercise: 25 | Diet:  32±1 (SE)  Diet+  exercise:  32±1 (SE) | Females | Mixed:  3*/w, 30-40 min cycling, HR that corresponded to an anaerobic threshold up to 10% below the respiratory compensation point and 20 min resistance and flexibility exercises  Supervision? | 600kcal/d energy restriction  (50-70C/10-15P/15-30F) | PP | DXA |
| Valsdottir  2020 | Norway | 2.5 | 57 | Diet:  15  Low-carbo-hydrate high-fat diet:  14  Diet+  exercise: 14  Low-carbo-hydrate high-fat diet+  exercise: 14 | Diet:  39±4  Low-carbohydrate high-fat  diet:  40±3  Diet+  exercise:  41±4  Low-carbohydrate high-fat  diet+  exercise:  41±3 | Females | Endurance:  3*/w, 7*4 min cycling intervals at 82-90% HRmax with 3 min rest at 60% HRmax | Diet:  700kcal/d calorie restriction  (45-60C/10-20P/25-40F)  Low-carbohydrate high-fat diet:  700kcal/d calorie restriction (5C/25P/70F) | ITT | DXA |
| Van Dale  1987 | Nether-lands | 2 | 12 | Diet:  6  Diet+  exercise:  6 | 29 (no group-specific data) | Females | Mixed:  4*/w with 2*/w endurance dancing sessions, 50-60% VO_2max_, 2*/w calisthenics & dancing, 50-60% VO_2max_ | Weeks 1-5: 680 kcal/d, weeks 6-13: 811 kcal/d, partly formula diet/meal replacement | PP | Hydrodensito-metry |
| Verreijen  2017 | Netherlands | 2.5 | 100 | Diet:  22  High-protein diet: 21  Diet+  exercise: 25  High-protein diet+  exercise: 32 | Diet:  63±4  High-  protein diet:  62±6  Diet+  exercise:  63±6  High-  protein  diet+  exercise:  62±5 | Males and females | Strength:  3*/w, 2-3 sets with  50-75 s per exercise, 9 exercises, supervised | Diet:  600kcal/d energy restriction with 0.8 g protein/kg body weight/d  High-protein diet;  600kcal/d energy restriction with 1.3 g protein/kg body weight/d | ITT | Air displacement plethysmo-graphy |
| Villareal  2011 | USA | 6 | 107 | Diet:  26  Diet+  exercise: 28 | Diet:  70±4  Diet+  exercise:  70±4 | Males and females | Mixed:  3*/w, 90 min sessions with cycling, treadmill walking, stair climbing, 65-85% HRmax and strength training with 1-3 sets, 6-12 reps, 65-80% 1-RM, supervised | 500-750 kcal/d energy restriction with 1g protein/kg body weight/d | ITT | DXA |
| Whatley  1994 | USA | 3 | 23 | Diet:  7  Diet+  exercise 3*/w:  8  Diet+  exercise 5*/w:  8 | Diet:  39±4  Diet+  exercise  3*/w:  39±7  Diet+  exercise  5*/w:  36±4 | Females | Mixed 3*/w:  3*/w, 30-70 min walking, 50-65% HR reserve and 2-3 sets, 6-8 reps, 70-80% 1-RM  Mixed 5*/w:  5*/w 30-80 min walking at 50-65% HR reserve and 3*/w 2-3 sets, 6-8 reps, 70-80% 1-RM  Both supervised | 803 kcal/d (55C/38P/7F), formula diet/meal replacement | PP | Hydrodensito-metry |
| Wood  2012 | USA | 3 | 42 | Diet:  8  Diet+  exercise:  9 | Diet:  58±7  Diet+  exercise:  59±6 | Males | Strength:  3*/w, 1-2 sets, 8-15 reps, 11 exercises, supervised | 1800 kcal/d with <30% of energy from fat | PP | BIA |
| Wycherley 2010 | Australia | 4 | 83 | Diet:  16  Diet+  exercise: 17  High-protein diet: 12  High-protein diet+  exercise: 14 | 51±10  (no group-specific data) | Males and females | Strength:  3*/w, 2 sets, 8-12 reps, 70-85% 1-RM, 8 exercises, supervised | Diet:  1434-1673 kcal/d (53C/19P/26F)  High-protein diet: 1434-1673 kcal/d (43C/33P/22F) | PP | DXA |

**Supplemental data file 3.** PEDro scoring details.

| **Study** | **Eligibility criteria** | **Random allocation** | **Concealed allocation** | **Baseline comparability** | **Blind subjects** | **Blind therapists** | **Blind assessors** | **Adequate follow-up**  **85%** | **Intention-to-treat analysis** | **Between-group comparisons** | **Point estimates and variability** | **Total score** |
| --- | --- | --- | --- | --- | --- | --- | --- | --- | --- | --- | --- | --- |
| Avila  2010 | 1 | 1 | 0 | 1 | 0 | 0 | 0 | 1 | 0 | 1 | 1 | 5 |
| Batitucci 2022 | 1 | 1 | 0 | 0 | 0 | 0 | 1 | 0 | 0 | 1 | 1 | 4 |
| Bhutani 2013 | 1 | 1 | 1 | 1 | 0 | 0 | 0 | 0 | 1 | 1 | 1 | 6 |
| Bouchard 2009 | 1 | 1 | 0 | 1 | 0 | 0 | 0 | 1 | 0 | 1 | 1 | 5 |
| Camajani 2022 | 1 | 1 | 0 | 1 | 0 | 0 | 0 | 1 | 1 | 1 | 1 | 6 |
| Cooke 2022 | 1 | 1 | 0 | 0 | 0 | 0 | 0 | 0 | 1 | 1 | 1 | 4 |
| Da Silva Soares 2022 | 1 | 1 | 0 | 1 | 0 | 0 | 0 | 1 | 1 | 1 | 1 | 6 |
| Deibert 2004 | 1 | 1 | 0 | 0 | 0 | 0 | 0 | 1 | 0 | 1 | 1 | 4 |
| Evans 2021 | 1 | 1 | 1 | 1 | 0 | 0 | 0 | 0 | 1 | 1 | 1 | 6 |
| Ezpeleta 2023 | 1 | 1 | 0 | 1 | 0 | 0 | 1 | 1 | 1 | 1 | 1 | 7 |
| Figueroa 2013 | 1 | 1 | 0 | 1 | 0 | 0 | 0 | 1 | 0 | 1 | 1 | 5 |
| Frimel 2008 | 1 | 1 | 0 | 1 | 0 | 0 | 1 | 1 | 0 | 1 | 1 | 6 |
| Geliebter 2014 | 1 | 1 | 0 | 1 | 0 | 0 | 1 | 0 | 0 | 1 | 1 | 5 |
| Hammer 1989 | 1 | 1 | 0 | 1 | 0 | 0 | 0 | 0 | 0 | 1 | 1 | 4 |
| Hosny 2012 | 1 | 1 | 0 | 1 | 0 | 0 | 0 | 1 | 1 | 1 | 1 | 6 |
| Janssen 1999 | 1 | 1 | 0 | 0 | 0 | 0 | 0 | 1 | 0 | 1 | 1 | 4 |
| Joseph 2020 | 0 | 1 | 0 | 1 | 0 | 0 | 0 | 0 | 0 | 1 | 1 | 4 |
| Kempen 1995 | 0 | 1 | 0 | 1 | 0 | 0 | 0 | 1 | 1 | 1 | 1 | 6 |
| Kleist 2017 | 1 | 1 | 0 | 1 | 0 | 0 | 1 | 1 | 0 | 1 | 1 | 6 |
| Kraemer 1999 | 0 | 1 | 0 | 1 | 0 | 0 | 0 | 0 | 0 | 1 | 1 | 4 |
| Mark 1995 | 1 | 1 | 0 | 1 | 0 | 0 | 0 | 0 | 0 | 1 | 1 | 4 |
| Miller 2020 | 1 | 1 | 1 | 1 | 0 | 0 | 0 | 0 | 1 | 1 | 1 | 6 |
| Nicklas 2009 | 1 | 1 | 0 | 1 | 0 | 0 | 1 | 1 | 1 | 1 | 1 | 7 |
| Relijc 2021 | 1 | 1 | 1 | 1 | 0 | 0 | 1 | 0 | 0 | 1 | 1 | 6 |
| Sañudo 2018 | 1 | 1 | 1 | 1 | 0 | 0 | 0 | 1 | 0 | 1 | 1 | 6 |
| Straznicky 2010 | 1 | 1 | 0 | 1 | 0 | 0 | 0 | 1 | 0 | 1 | 1 | 5 |
| Trombetta 2003 | 0 | 1 | 0 | 1 | 0 | 0 | 0 | 1 | 1 | 1 | 1 | 6 |
| Valsdottir 2020 | 1 | 1 | 0 | 1 | 0 | 0 | 0 | 1 | 1 | 1 | 1 | 6 |
| Van Dale 1987 | 0 | 1 | 0 | 1 | 0 | 0 | 0 | 0 | 0 | 1 | 1 | 4 |
| Verreijen 2017 | 1 | 1 | 1 | 1 | 0 | 0 | 0 | 0 | 1 | 1 | 1 | 6 |
| Villareal 2011 | 1 | 1 | 0 | 1 | 0 | 0 | 0 | 1 | 1 | 1 | 1 | 6 |
| Whatley 1994 | 1 | 1 | 0 | 1 | 0 | 0 | 0 | 1 | 1 | 1 | 1 | 6 |
| Wood 2012 | 1 | 1 | 0 | 1 | 0 | 0 | 0 | 0 | 0 | 1 | 1 | 4 |
| Wycherley 2010 | 1 | 1 | 0 | 1 | 0 | 0 | 0 | 0 | 1 | 1 | 1 | 5 |
|  |  |  |  |  |  |  |  |  |  |  |  | **MEAN=5.3**  **SD=1.0** |

**Supplemental data file 4.** RoB2 scoring details.


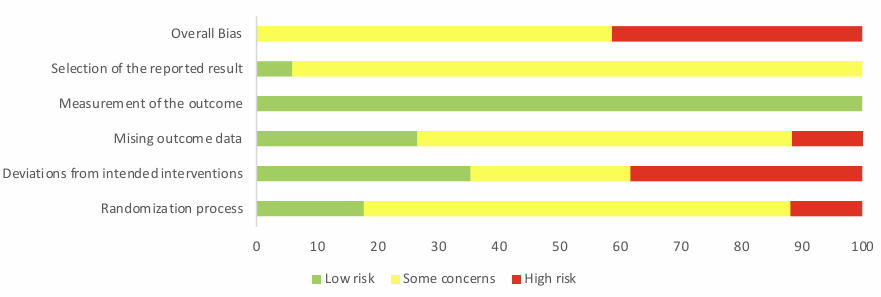


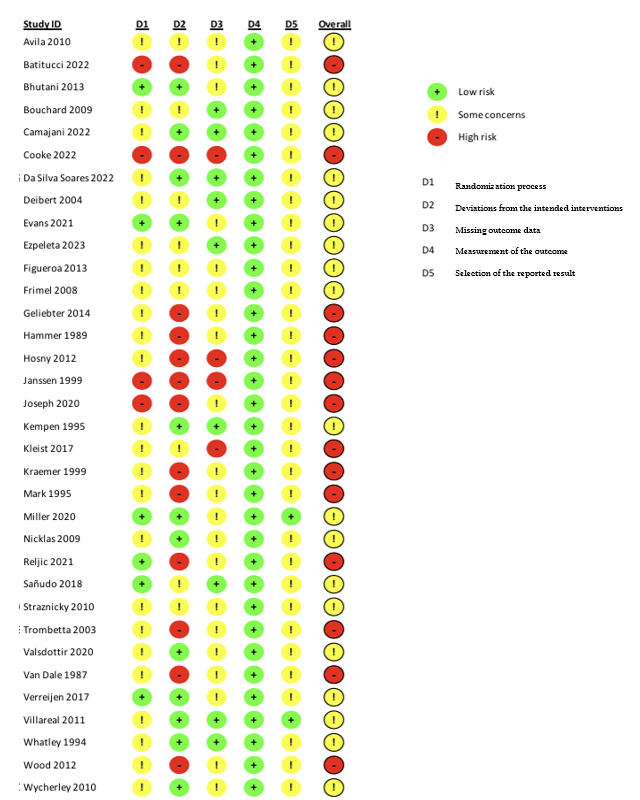


**Supplemental data file 5.** Meta regression analyses.


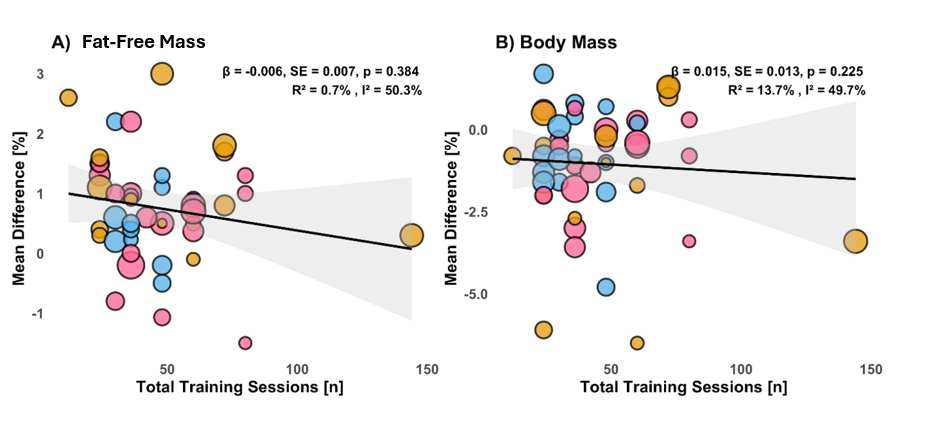


**Figure S1.** Meta regression of the intervention effect as a function of the total number of exercise training sessions. Panel A presents results for fat-free mass (FFM), and Panel B presents results for body mass. Points represent individual study effects weighted by total sample size, with colours indicating mode of training. Solid lines denote fitted meta regression slopes, and shaded areas represent 95% confidence bands. Each panel reports the regression coefficient, standard error, p value, and the proportion of heterogeneity explained (R²).

**Supplemental data file 6.** Funnel plots.


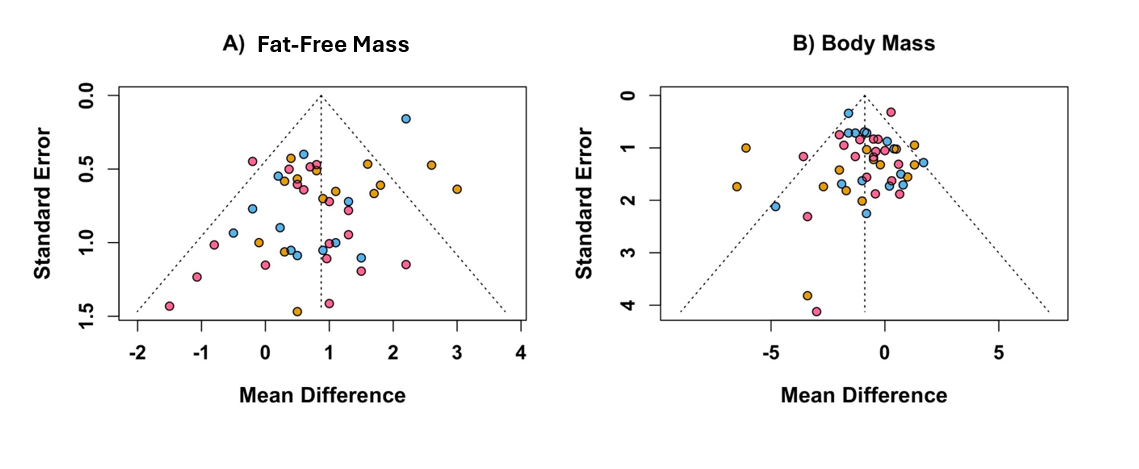


**Figure S2.** Funnel plots for the meta-analyses of fat-free mass (FFM) (A) and body mass (B). Each point represents an individual study effect plotted against its standard error, with the vertical line indicating the pooled random effects estimate and diagonal lines representing the expected pseudo confidence limits. Colours denote mode of training (orange = strength, blue = mixed, red = endurance).
